# Rapid lipid-laden plaque identification in intravascular optical coherence tomography imaging based on time-series deep learning

**DOI:** 10.1117/1.JBO.27.10.106006

**Published:** 2022-10-28

**Authors:** Jose J. Rico-Jimenez, Javier A. Jo

**Affiliations:** a Texas A&M University, Department of Biomedical Engineering, College Station, Texas, United States; b University of Oklahoma, School of Electrical and Computer Engineering, Norman, Oklahoma, United States

**Keywords:** intravascular optical coherence tomography, automated plaque assessment, deep-learning-based lipid-laden plaques identification

## Abstract

**Significance:**

Coronary heart disease has the highest rate of death and morbidity in the Western world. Atherosclerosis is an asymptomatic condition that is considered the primary cause of cardiovascular diseases. The accumulation of low-density lipoprotein triggers an inflammatory process in focal areas of arteries, which leads to the formation of plaques. Lipid-laden plaques containing a necrotic core may eventually rupture, causing heart attack and stroke. Lately, intravascular optical coherence tomography (IV-OCT) imaging has been used for plaque assessment. The interpretation of the IV-OCT images is performed visually, which is burdensome and requires highly trained physicians for accurate plaque identification.

**Aim:**

Our study aims to provide high throughput lipid-laden plaque identification that can assist *in vivo* imaging by offering faster screening and guided decision making during percutaneous coronary interventions.

**Approach:**

An A-line-wise classification methodology based on time-series deep learning is presented to fulfill this aim. The classifier was trained and validated with a database consisting of IV-OCT images of 98 artery sections. A trained physician with expertise in the analysis of IV-OCT imaging provided the visual evaluation of the database that was used as ground truth for training and validation.

**Results:**

This method showed an accuracy, sensitivity, and specificity of 89.6%, 83.6%, and 91.1%, respectively. This deep learning methodology has the potential to increase the speed of lipid-laden plaques identification to provide a high throughput of more than 100 B-scans/s.

**Conclusions:**

These encouraging results suggest that this method will allow for high throughput video-rate atherosclerotic plaque assessment through automated tissue characterization for *in vivo* imaging by providing faster screening to assist in guided decision making during percutaneous coronary interventions.

## 1 Introduction

Heart disease is the leading cause of death in Western society. In the United States, heart disease is linked to 1 of 5 deaths, for which the medications and healthcare procedures result in an economic impact of hundreds of billions of dollars each year.^1^ The accumulation of lipids and fibrous elements in the arteries is the main contributor to cardiovascular disease. Stable plaques are characterized by the accumulation of collagen that forms a fibrous plaque, which can cause progressive stenosis, whereas vulnerable plaques result from the buildup of lipids and necrotic core, which in turn can lead to sudden thrombosis and heart attack.^2^ An abnormal buildup of lipids in the artery is known to initiate atherogenesis by eliciting an inflammation response that ultimately makes the plaque vulnerable to erosion and rupture.^3^^,^^4^

Intravascular optical coherence tomography (IV-OCT) provides detailed high-resolution imaging of the microstructure information of coronary plaques,^5^^–^^7^ where the intima, media, and adventitia layers can be differentiated. Detecting lipid-laden plaques prone to rupture using IV-OCT imaging, especially accompanied by macrophages and necrotic core, is clinically relevant.^2^^,^^8^^,^^9^ Therefore, detecting lipid-rich plaques during PCI could in the future enable the selection of the best stent or local therapy for both culprit and specific nonculprit plaques and allow for guiding systemic therapy based on the entire coronary tree lipid burden.^10^ However, visual interpretation of full optical coherence tomography (OCT) pullbacks with hundreds of B-scans is tedious and extremely time-consuming, and it requires a trained eye to evaluate different features related to vulnerable plaque development due to speckle and scattering attenuation effects.^11^^,^^12^ The effect of speckle noise in IV-OCT image interpretation has not been systematically quantified; however, speckle noise is ubiquitous in OCT imaging. Speckle noise deteriorates the image quality by reducing contrast and producing blurry details in local features; thus it can preclude the accurate visual interpretation of OCT images. There have been significant research efforts focused on developing image processing strategies for reducing speckle noise to enable more accurate OCT image interpretation. Neural network models, on the other hand, in principle, can learn relevant image features even when trained with noisy data. Thus it is expected that neural network models trained for plaque classification will be more robust to speckle noise in IV-OCT imaging data.

The use of convolutional neural networks (CNNs) in the medical imaging field has increased significantly. Multiple studies have focused on automatically identifying different tissue categories in OCT images using CNN, mainly for retinal imaging.^13^^–^^15^ Recently, some efforts to classify different types of plaques using deep learning have been reported.^16^^,^^17^ Plaque detection of full IVOCT pullbacks using predesigned ResNet and DenseNet CNNs was demonstrated.^18^ This method requires preprocessing that involves extreme image resizing from 1920 pixels to 300 pixels and exhaustive shifting and rotating of the images for data augmentation. An approach for characterization of coronary arteries from OCT imaging based on deep learning using pretrained CNN was proposed.^19^ This study reports high values of accuracy, sensitivity, and specificity for different tissue categories; however, it was tested with a small database of only 33 cases, which may lead to biased performance because an adequate cross-validation strategy was not applied. Moreover, it requires a majority voting strategy to achieve robust automatic interpretation of OCT images. He et al.^20^ used CNN-based pixel classification for automated plaque characterization of OCT images, where the plaque area was extracted through lumen border detection and expansion. The plaque area was classified as lipid tissue, fibrous tissue, mixed tissue, calcified tissue, or background with an average accuracy of 86.6%. Lee et al.^21^ presented a deep learning technique for automated segmentation of plaques components in which a predesigned CNN classifier (SegNet) was used. Although it reported a high sensitivity for detecting lipids of 87%, agreeing with the manually annotated counterparts, this method requires preprocessing, segmentation, and postprocessing classification noise-reduction tasks that reduce the processing time of entire pullbacks to 300 to 500 frames in 80 to 135 s, which may not be suitable for *in vivo* clinical interventions. The same research group developed A-line plaque classification approaches that reported similar sensitivity and the need for extensive preprocessing, feature extraction, classification, and classification noise-cleaning tasks.^22^^,^^23^ Later, they used a robust hybrid learning approach that combined deep-learning convolutional and hand-crafted morphological features.^24^ This method depends on the conditional random field for postprocessing to reduce classification errors. Abdolmanafi et al.^19^^,^^25^ presented two deep-learning-based approaches to discriminate coronary artery layers and plaques, in which a pretrained CNN model (AlexNet) was utilized as a feature generator and random forest and support vector machine models were used for classification and segmentation, respectively. Another method presented by Gessert et al.^18^ combined the pretrained ResNet50-V2 and DenseNet121 for binary plaque characterization. This combined approach achieved an accuracy of 91.7%. Another deep-learning-based approach for plaque characterization of four plaque types (lipid tissue, fibrous tissue, calcification, and mixed tissue) reported an average classification accuracy of 86.6% for all classes.^20^ However, the performance for lipid tissue and mixed tissue identification was only 60.5% and 66.6%, respectively. Cheimariotis et al.^26^ proposed a CNN that takes attenuation-coefficient IV-OCT B-scans as input to classify individual A-lines. This method requires several processing steps such as image resizing, noise removal, wall segmentation, and attenuation coefficient estimation. The results showed slightly higher accuracy when using the attenuation coefficient. Although this method showed high sensitivity of 87.78% when discriminating between plaque and normal A-lines, the specificity of 61.45% was low compared with similar approaches. In our previous work,^27^ we demonstrated a plaque tissue characterization methodology based on intrinsic morphological characteristics of the A-lines using OCT imaging to classify intimal-thickening, fibrotic, superficial-lipid, and fibrotic-lipid by applying linear discriminant analysis. However, this method requires image preprocessing and specific feature extraction, which may require complex parallel algorithms for *in vivo* imaging of large OCT pullbacks.

In recent years, time-series forecasting using CNNs has gained popularity for scientific and industrial applications that require analysis of continuous data such as climate, biological sciences, and finance.^28^ In time-series forecasting, sliding window filters are applied to sequential data, and the learned features are flattened as input to the fully connected layers.^29^ Our premise is that pixels in an OCT A-line can be used as sequential data in a CNN-time-series classifier. Thus the consecutive pixels in the depth of each A-line can be used as sequential data in time. Therefore, this paper reports the capability of a simple and computationally efficient deep learning method based on time series for automated, accurate, and fast identification of lipid-laden plaques in IV-OCT images of coronary arteries. A sequential CNN model composed of one-dimensional convolutional and dense layers is used for binary classification. This approach does not require heavy preprocessing, data augmentations, or feature crafting because the CCN works as an automated feature extractor. Importantly, the use of appropriate parallel processing hardware and libraries will allow for achieving B-scan video-rate lipid identification.

## 2 Materials and Methods

### 2.1 Database

**Fig. 1:**
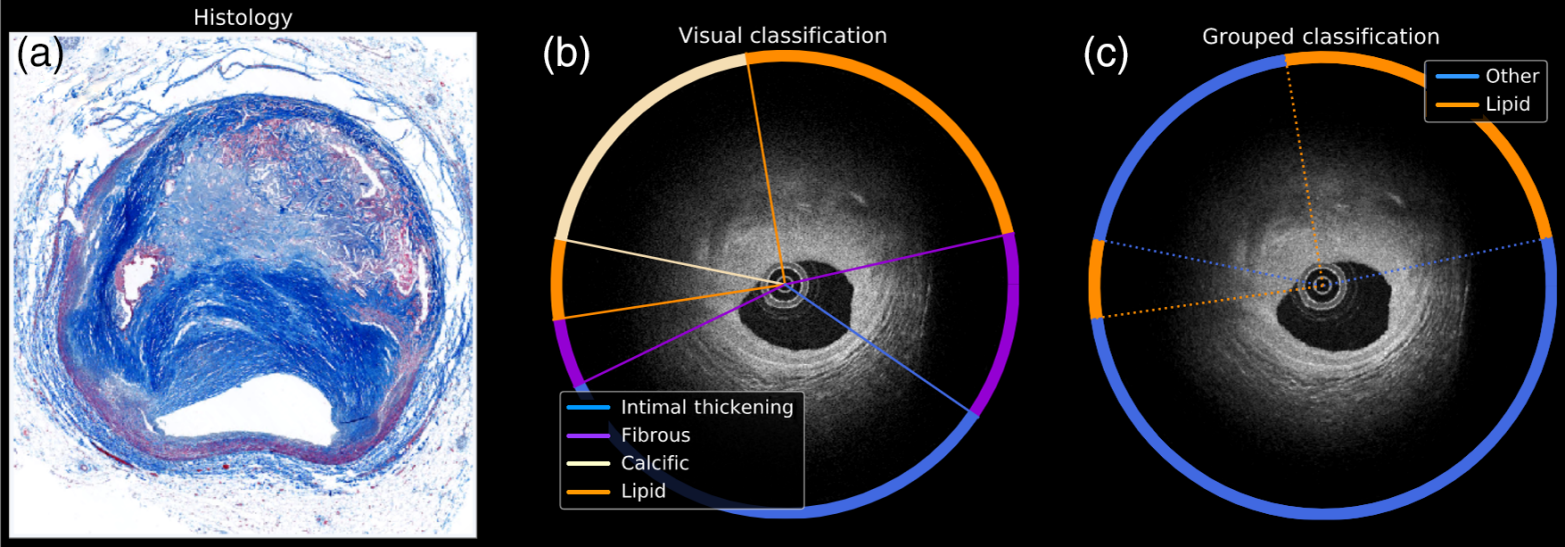
Visual evaluation of the IV-OCT B-scans. (a) Masson’s Trichrome histological image as reference. (b) Visual classification overlaid on the IV-OCT image. (c) Grouped version of the visual classification with only the “lipid” and “other” categories.

The database consists of a collection of 98 IV-OCT images from 10 coronary arteries of five cadaveric human hearts. The imaging protocol was previously described in Ref. 30. The images were acquired using a custom swept-source polarization-sensitive OCT (PS-OCT) microscope (swept range of 115 nm centered at 1320 nm; axial resolution of 9.4  μm in tissue, assuming a refractive index of 1.34; and repetition rate of 54 kHz).^30^^–^^32^ Each IV-OCT B-scan consists of 1024 A-lines (angular sampling step of 0.35 deg) with 1024 pixels along the depth (axial sampling step of 4.8  μm), acquired during volumetric imaging at a pullback speed of 5 mm/s. This imaging system acquires two orthogonal polarization channels in parallel alternating the polarization state of the light incident on the sample between A-lines. Although the OCT images were acquired using an intravascular PS-OCT prototype system, focusing on standard IV-OCT imaging data analysis (i.e., not considering the polarization states of the OCT signal) will have a greater, more immediate impact as intravascular PS-OCT is not yet being used clinically. Therefore, for this study, the sum of the squared norm of the complex-valued tomogram of each channel was computed, ignoring the modulation of the polarization states between A-lines. A total of 98 IV-OCT pullback scans were acquired from 10 coronary trees. Each pullback dataset consisted of 9 B-scans; however, matching histology sections were only available for the center B-scan of each pullback. Because only one B-scan with a matching histology section was available from each pullback, the dataset used in this study includes a total of 98 IV-OCT B-scans with matching histology sections. None of the B-scans were consecutive from the same plaque or artery segment. Therefore, the training and testing sets were composed of different B-scans, with none of them being consecutive from the same plaque or artery segment. The ground truth for each B-scan was obtained as follows. An expert IV-OCT reader provided an initial visual annotation of each B-scan [Fig. 1(b)]. Then the visual annotation was reviewed against the corresponding available histology section [Fig. 1(a)] and revised as needed. The visually assessed and histologically confirmed ground truth of each B-scan was finally simplified into a binary (lipid versus other) annotation [Fig. 1(c)]. The expert OCT reader (K. O.) visually reviewed each B-scan and classified each A-line into one of five categories based on established OCT criteria based on consensus standards provided and published by the International Working Group for Intravascular Optical Coherence Tomography (IWG-IVOCT) Standardization and Validation: (i) intimal-thickening, (ii) fibrous, (iii) calcific, (iv) lipid, and (v) guidewire. A custom MATLAB software allowed the expert observer to visually examine the B-scans and mark the lumen surface by defining arcs as depicted in Fig. 1. Each A-line was labeled with one of the categories mentioned above to describe the most dominant class [Fig. 1(b)]. The resulting labels were saved in individual files for each sample. Because the purpose of this study is the identification of lipid-laden plaques, all other categories different from lipid were grouped into a single category named “other” [Fig. 1(c)].

The formation of lipid plaques involves monocytes infiltration in the intima. These monocytes differentiate into macrophages to ingest lipids.^4^^,^^33^ Subsequently, lipid-laden macrophages known as foam cells will eventually die, contributing to the establishment of a necrotic core in the plaque.^9^ Identification of lipid-laden plaques accompanied by necrotic cores would be clinically relevant. However, to our knowledge, IV-OCT has not been able to differentiate between these two categories. Therefore, the lipid category implicitly includes lipids, macrophage/foam cells, and necrotic core. Moreover, some plaques that contain a mix of lipids and fibrous tissue are marked with the most dominant class.

### 2.2 Data Processing

An advantage of the proposed approach is the minimalistic image processing required. The linear OCT images are converted to their log-compressed version and downsampled to a half (512×512  pixels) to increase the signal-to-noise ratio and reduce the computational load; however, the high-resolution image of 1024 pixels can still be used for visualization with upsampled classification. Because the standardization of datasets is a critical step in neural network models,^34^^,^^35^ the data are standardized using the scale function of Scikit-learn to accelerate and facilitate the convergence of the estimator. No other image processing operation is needed. Arrays of A-lines are fed directly into the CNN model, resulting in arrays of predictions.

### 2.3 Training, Testing, and Validation

**Fig. 2:**
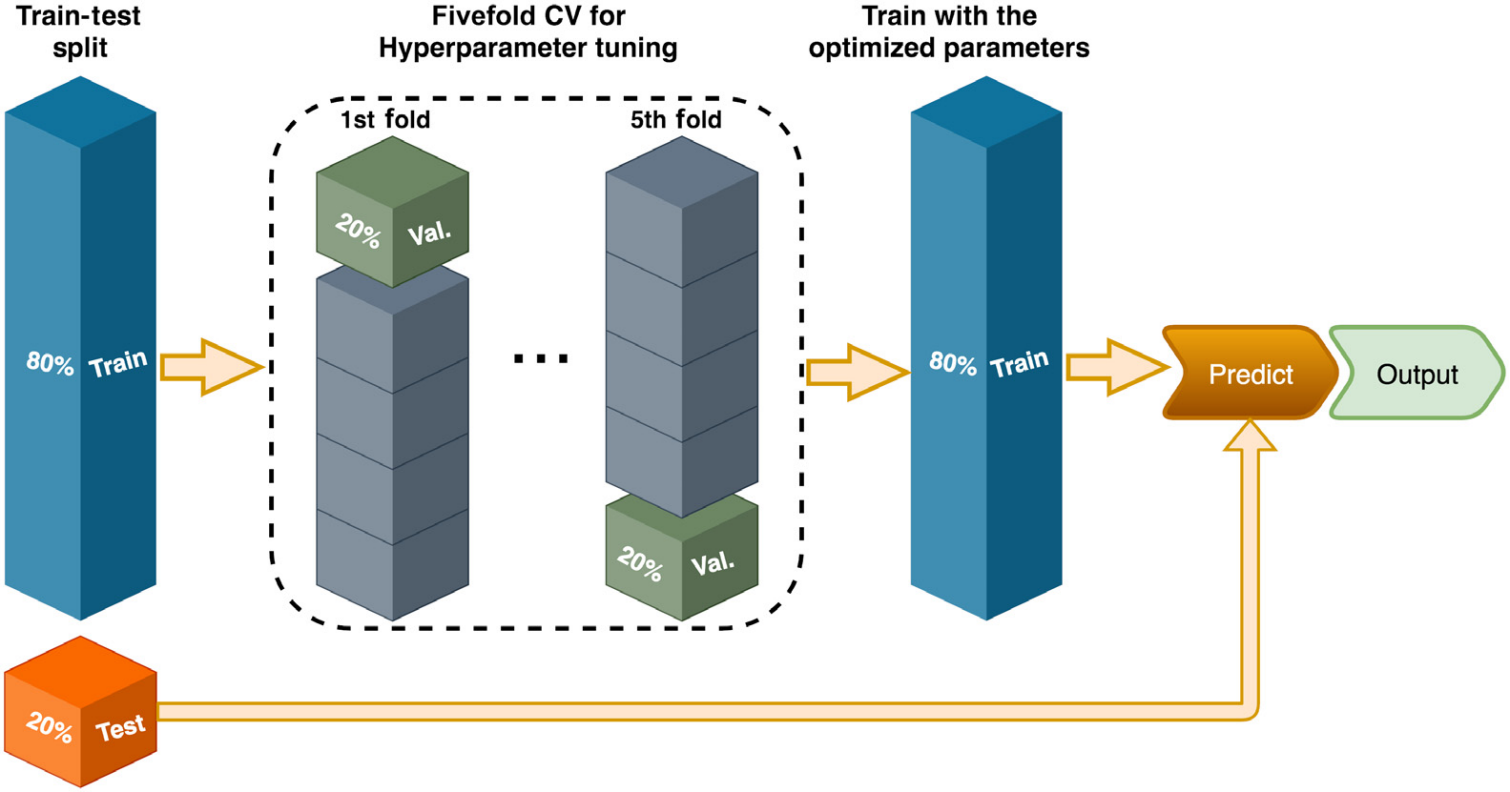
Data stratification for training, testing, and validation following a fivefold strategy. The whole database is randomly shuffled and then divided into 80% for training and ∼20% for testing. Likewise, in the hyperparameter tuning task, the training set is split into 5 blocks of 20%, where each block in turn serves as validation and the rest of the data is used for training. The testing set is fed into the model to predict the presence of lipids in the A-line.

The proposed binary classifier was then trained and validated using only the lipid and other categories. The database was randomly shuffled and split into ∼80% (78 B-scans) for training and ∼20% (20 B-scans) for testing the CNN. It is worth noting that none of the B-scans are consecutive from the same plaque or artery segment. Additionally, the training set was divided 5 times into ∼80% (62 B-scans) for training and ∼20% (16 B-scans) for validation to follow a fivefold cross validation used for fine-tuning the hyperparameters. The training–testing–validation strategy is illustrated in Fig. 2.

**Fig. 3:**
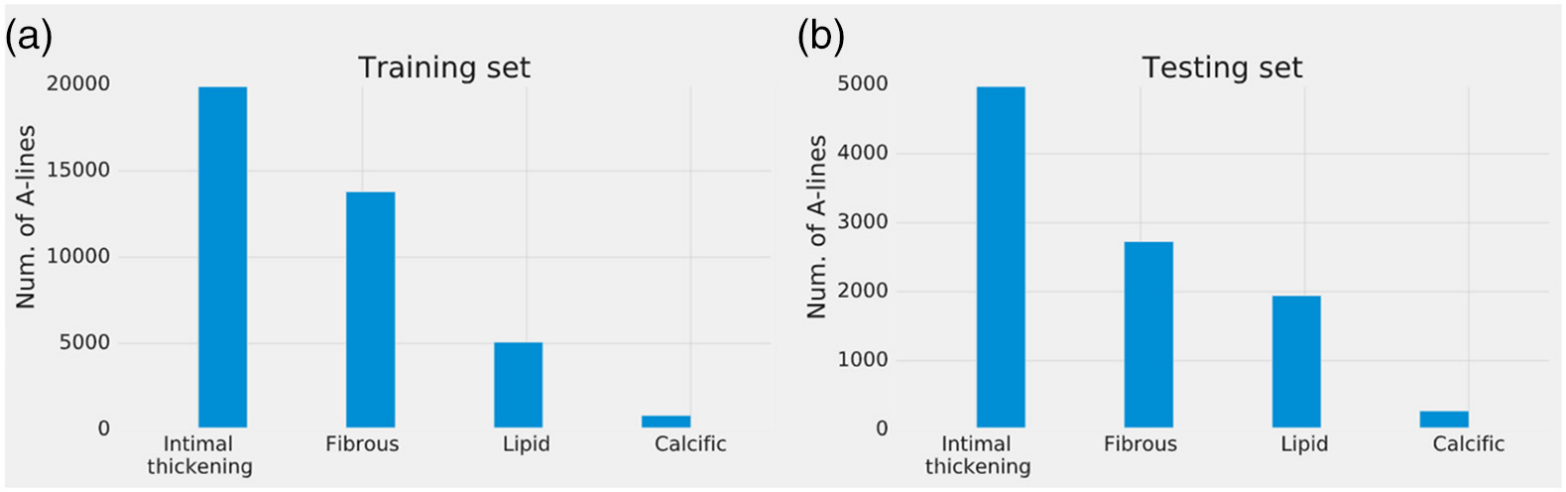
A-line distribution of the (a) training and (b) testing sets from the visual assessment.

The A-line class count in the database is imbalanced. Figure 3 shows the A-line count distribution of each category for the training and testing sets. Therefore, an algorithm from the Scikit-learn library was used to estimate class weights for the imbalanced dataset that is based on the balanced heuristic used in logistic regression.^36^

### 2.4 Convolutional Neural Network Design

**Fig. 4:**
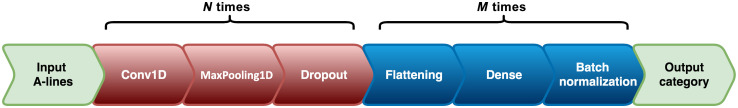
Neural network architecture. The first block consists of a combination of Conv1D, MaxPooling1D, and dropout layers. The second block is formed by flattening, fully connected dense, and batch-normalization layers. The first and second blocks may repeat N and M times, respectively.

The CNN was designed based on time-series deep learning, which has been successfully implemented for the classification of sequential data, such as audio signals for speech recognition. In the particular case of OCT, A-lines represent sequential data in depth. Therefore, in the proposed approach, temporal ordering between data points was replaced by depth-spatial ordering between pixels in the A-line. The topology of the CNN is graphically described in Fig. 4. The CNN consists of a stack of sequential layers with two blocks that can repeat multiple times. The first block is intended to pick the most relevant features related to differences in signal attenuation in the A-line. The second block is intended to use the extracted features in the first block to find differences between A-lines belonging to different classes.

In the first block of layers, the one-dimensional spatial convolutional layer (Conv1D) performs the convolution along the A-line or depth dimension. Using Conv1D layers, the model extracts the features from sequential data regardless of where the feature is located in depth. The Maxpooling operation (MaxPooling1D) is used after Conv1D to downsample the input representation by keeping only the maximum value over the window, which reduces the computational cost by reducing the number of parameters to fit. Likewise, a dropout operation is used to reduce overfitting and refine the generalization of the CNN. In the second block, the flattening operation creates a single feature vector that serves as input for the fully connected dense layers. Each dense layer is followed by batch normalization and dropout operations, which dramatically accelerate the training process of the neural network.^34^ The output layer is a dense layer with two units and sigmoid activation functions. Strategies of early stopping and reducing the learning rate on a plateau were followed to stop training once the model performance stops improving and to converge to new minima by taking smaller learning steps, respectively. Additionally, the training was automatically stopped if the number of epochs reached the maximum of 100. On average, the training task stopped at ∼50 epochs. The model was fit using a binary cross-entropy loss function that is minimized during training.

## 3 Results

The CNN model was implemented in Python using the Keras library with TensorFlow as the backend running on a computer with Windows 7 Enterprise 64-bit on an Intel i7 CPU with 6 cores at 3.2 GHz and with 48 GB RAM.

### 3.1 Hyperparameter Tuning

**Fig. 5:**
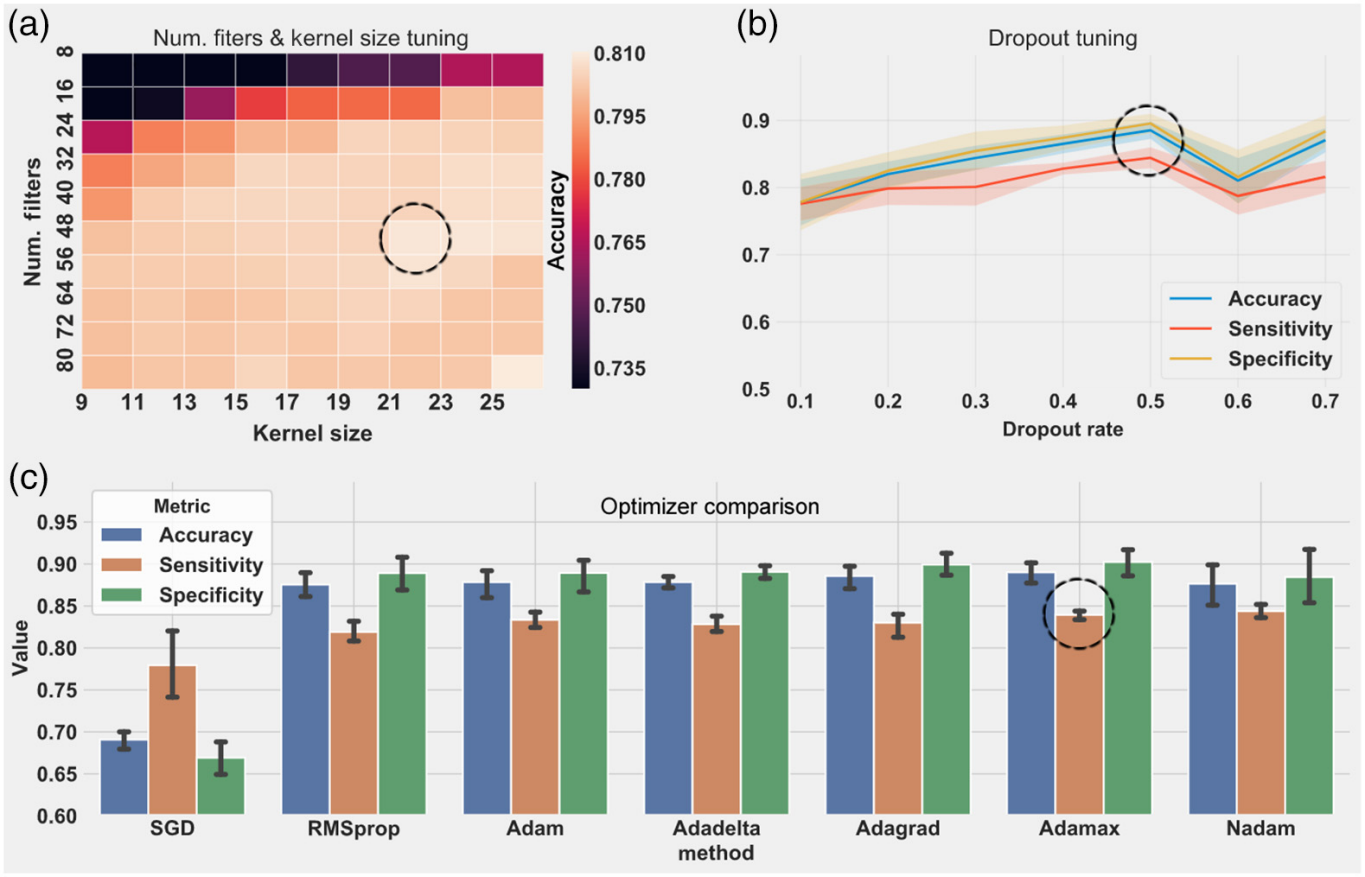
Examples of the CNN hyperparameter tuning. (a) Accuracy variations during tuning of the number of filters and kernel size of the Conv1D layer. (b) Dropout tuning in the first block of the CNN. (c) Performance of different optimizers. Dashed circle points at best performance.

A fivefold cross-validation strategy was followed to optimize the hyperparameters. The training set was split into five chunks of 20% of the data, where one chunk was used for validation and the rest of the data was used for training; this process was repeated 5 times. At each iteration, the Keras backend session was cleared and reinitialized to avoid clutter from the model of the previous iteration. Layers in the CNN are computationally represented as functions with none or multiple arguments or parameters. First, the CNN was evaluated with only default parameters. Then, for each layer with one or more parameters, each parameter was fine-tuned by assigning a range of values, and the average classification performance for the fivefolds was recorded. Figure 5 shows trends in CNN performance during the hyperparameter tuning task. As shown in Fig. 5(a), the Conv1D number of filters and kernel size showed the best performance at 48 and 21, respectively. The performance increases with the dropout rate until a rate of 0.5. It is not recommended to use a dropout rate higher than 0.5 because the CNN becomes unstable, as illustrated in Fig. 5(b); thus the optimal dropout rate was identified as 0.5. Figure 5(c) shows the performance of different optimizers. Although Adamax and Adagrad showed the best performances, Adamax was selected because it had a slightly better accuracy and sensitivity. Although Adamax and Adagrad show similar performances for different applications, implementing Adamax is straightforward and requires less memory.^37^

The result of the tuning task is summarized in Table 1. The input layer receives an array of size S×512  pixels containing a batch of S number of A-lines of 512 pixels in depth. For Conv1D, three identical layers (N=3; levels 1, 4, and 7) with 48 filters of size 21 and an activation rectified linear activation function (ReLU) are implemented. The MaxPooling1D optimal pool size was 2, and the dropout rate was 0.5. For the last block, the output was flattened (level 10). A dense layer of 16 (level 11) units with activation ReLU and disabled bias (required for batch normalization) was used. The output dense layer returns an array of size S with the binary classification results.

**Table 1:** Final configuration of the CNN.

Layer	Level	Parameter description
Input	0	Array of S A-lines: any number of downsampled A-lines (512 pixels depth)
Conv1D	1, 4, 7	Filters: 48; kernel size: 21; padding: “same;” activation: ReLU; strides: 1
MaxPooling1D	2, 5, 8	Pool size: 2.
Dropout	3, 6, 9	Rate: 0.5
Flattening	10	
Dense	11	Units: 16, 8; activation: ReLU; and use bias: false
Batch normalization	12	
Dense (output)	13	Units: 2; activation: sigmoid; output: array of binary class predictions for the input array of A-lines. 0: no-lipid; 1: lipid

### 3.2 Validation

After fine-tuning the hyperparameters, the initial split of 80% of the whole database was used to retrain the network with the optimized parameters. The remaining 20% of data that was reserved for testing was then fed into the retrained CNN model to predict the presence of lipids in the test A-lines.

**Fig. 6:**
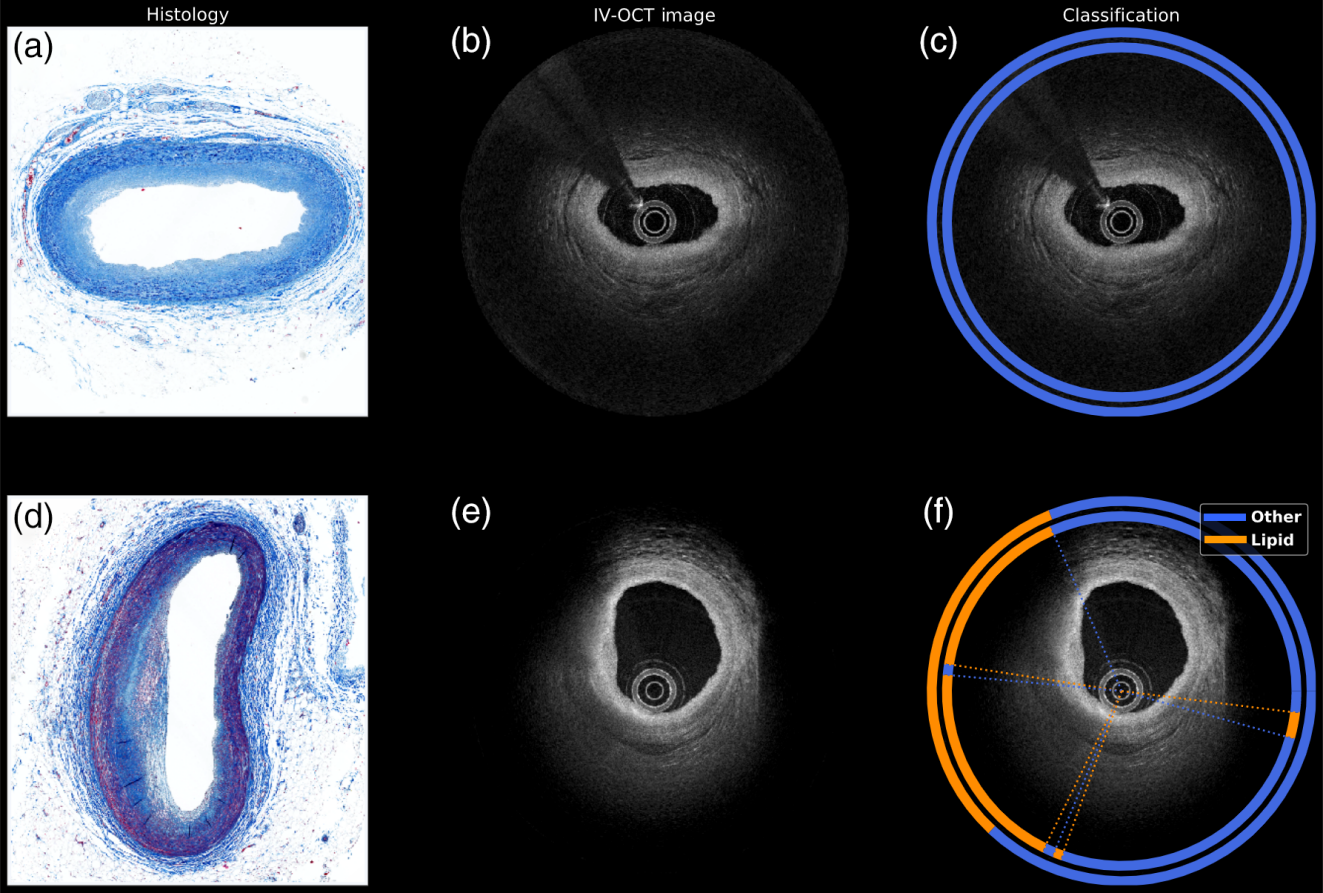
Automated lipid classification of two samples in the testing set. (a), (d) Masson’s trichrome histological images; (b), (e) original OCT image; and (c), (f) classification maps. (a)–(c) A fibrous plaque with a guidewire artifact and (d)–(f) a lipid plaque. The outer ring denotes the visual classification (ground truth), and the inner ring indicates the automated classification (orange is lipid and blue is other) (Video 1, MPG, 0.742 MB [URL: https://doi.org/10.1117/1.JBO.27.10.106006.s1]; Video 2, MPG, 0.790 MB [URL: https://doi.org/10.1117/1.JBO.27.10.106006.s2]).

Because the neural network is initialized with random weights, it leads to different starting points per each simulation during the training phase and, therefore, a slightly different outcome at each training run. Consequently, the last training–testing task was repeated 10 times by randomly shuffling the database and then dividing it into 80% for training and 20% for testing at each iteration. The algorithm reported an accuracy, sensitivity, and specificity of 89.6±0.01%, 83.6±0.01%, and 91.1±0.01%, respectively. The results of the classification for two representative IV-OCT B-scans are graphically depicted in Fig. 6. The outer ring overlaid on the IV-OCT images (c) and (f) shows the visual classification, and the inner ring shows the automated classification (orange: lipid and blue: other). Figures 6(a)–(c) show a fibrous plaque with a guidewire artifact, where both the visual and automated classifications identified a plaque without lipid presence. Figures 6(d)–(f) show a mixed lipid–fibrous plaque, where the visual and automated classification matched ∼90%. Examples of the classification of full pullbacks are illustrated in Videos 1 and 2.

Image processing and classification times are critical for *in vivo* image-guided interventions. In terms of prediction time, the CNN classification performance was quantified for different computation scenarios, as summarized in Table 2. The final trained model was saved in a file and then loaded to be used by another script to measure the prediction performance on the testing set (20 B-scans) running in two different hardware configurations. Computer 1, used for designing and training the CNN, did not have GPU capability. Therefore, the model was also tested in another computer with GPU capability (computer 2). The average CPU prediction rate of computer 1 was ∼13 B-scans/s. Likewise, the average CPU classification rate of computer 2 was only ∼8 B-scans/s. On the other hand, as expected, the average GPU prediction rate of computer 2 was ∼104 B-scans/s, showing superior classification throughput.

**Table 2:** CNN Classification rate running on two different hardware configurations.

	Computer 1	Computer 2	
	CPU	CPU	GPU
Hardware description	Intel^®^ Core™ i7-3930k CPU at 3.2 GHz; 6 cores. 48 GB RAM	Intel^®^ Core™ i7-4820k CPU at 3.7 GHz; 4 cores. 64 GB RAM	GeForce GTX TITAN X with compute capability: 5.2
Classification rate (B-scans/s)	∼13.5	∼8.2	∼104.6

## 4 Discussion

IV-OCT plaque characterization requires trained experts to visually evaluate each B-scan, which is cumbersome and complicated due to variation in the tissue attenuation and speckle effects.^16^^,^^17^ Blood cells cause light scattering, which can degrade the image quality and impair OCT image interpretation. During IV-OCT imaging, blood is displaced by injecting a saline solution into the coronary ostium; nevertheless, *in vivo* IV-OCT images can still suffer from image degradation due to residual blood speckle artifacts. Thus a predictive model suitable for clinical use will have to be trained using clinical IV-OCT data that incorporate the expected typical noise sources and artifacts while imaging in the catheterization lab. It should be noted that neural network models can, in principle, learn relevant image features even when trained with noisy data. Thus it is expected that neural network models trained with clinical IV-OCT data will be more robust to blood speckle image degradation. In practice, IV-OCT imaging of atherosclerotic plaques requires pullbacks consisting of hundreds of B-scans, making visual plaque classification unfeasible in clinical settings. Therefore, we present a computationally efficient IV-OCT classification method that could offer rapid automated fast identification of lipids in coronary atherosclerotic plaques. This binary classification method makes use of the one-dimensional CNN architecture to discriminate A-lines within lipid-laden plaques.

The association between calcified plaques and plaque vulnerability is unclear. In most cases, coronary thrombosis is caused by plaque rupture or plaque erosion, whereas calcified nodules are rarely present.^2^^–^^5^ The database used in this study contains limited calcified plaque cases (see Fig. 3); therefore, the models developed using this limited database would not be optimal for discriminating lipid rich from calcified plaques. Nevertheless, more optimal DL models can be trained using more comprehensive IV-OCT databases following the strategy applied in this study.

The main limitation of this study is the limited size of the data set used. This is a common problem in the field, as IV-OCT databases with ground truth based on histopathology are restricted to *ex vivo* (postmortem) imaging and limited in size. Therefore, developing prediction models for plaque classification using comprehensive datasets with histopathology ground truth is unfeasible. Nevertheless, the small IV-OCT dataset with both histopathology and visual assessment ground truth that was available for this study enabled exploring the use of neural network models for predicting lipid-rich plaques from IV-OCT images. Future efforts will focus on demonstrating the feasibility of developing prediction models for plaque classification using IV-OCT datasets with only visual assessment ground truth.

Another limitation of this study is the use of manually annotated images by only one trained expert in IV-OVT as ground truth. Additionally, labeling should come from a histological assessment, which would be the ideal gold standard. However, histological images are not available in clinical scenarios where *in vivo* IV-OCT imaging is practiced. Thus plaque assessment relies only on visual examination. We demonstrated that our method can mimic the visual interpretation of the IV-OCT images, which is imperfect due to the difficulty of resolving features due to speckle and attenuation effects. Nevertheless, the use of more accurate labeling provided by more than one observer might increase the outcome of this method.

A specific model trained with the limited IV-OCT data available does not provide depth classification; thus it will not enable distinguishing between thin versus thick fibrous caps. However, neural network models should be able to pick A-line features that might lead to the discrimination between thin-cap versus thick-cap fibroatheroma if these models can be trained with sufficiently large and comprehensive databases of IV-OCT scans.

Most deep learning approaches use data augmentation to expand the size of a training dataset by creating altered versions of the data and increasing the volume of relevant data in the dataset. This study demonstrated a minimalistic deep learning approach that can detect lipids in IV-OCT images without data augmentation. Nevertheless, a larger database will help to increase the generalization and, therefore, the accuracy of the CNN model. Furthermore, a larger database with a higher generalization of plaques allows for the classification of calcific plaques and the differentiation between intimal-thickening and fibrous plaques.

This approach may lead to real-time IV-OCT assessment of a complete pullback and thus the possibility of (near) real-time assessment of the entire dataset of a patient undergoing PCI. This would be feasible with current/future GPU capabilities for deep learning inference (e.g., the network can be optimized for higher throughput using network pruning techniques) and visualization. Complementing this classification methodology with advanced visualization and analysis techniques will certainly be relevant for longitudinal tracking of disease progression and/or treatment response. The expected computational speed of a fully trained, optimized, and validated model will enable near real-time visualization of lipids in both cross-sectional and 2D maps of full pullbacks, improving decision making during PCI.

## Code, Data, and Materials Availability

Data underlying the results presented in this paper are not publicly available at this time but may be obtained from the corresponding author upon reasonable request.
